# Performance Assessment of Multistatic/Multi-Frequency 3D GPR Imaging by Linear Microwave Tomography

**DOI:** 10.3390/s25206467

**Published:** 2025-10-19

**Authors:** Mehdi Masoodi, Gianluca Gennarelli, Carlo Noviello, Ilaria Catapano, Francesco Soldovieri

**Affiliations:** Institute for Electromagnetic Sensing of the Environment, National Research Council of Italy, Via Diocleziano 328, 80124 Napoli, Italy; gennarelli.g@irea.cnr.it (G.G.); noviello.c@irea.cnr.it (C.N.); catapano.i@irea.cnr.it (I.C.); soldovieri.f@irea.cnr.it (F.S.)

**Keywords:** ground-penetrating radar, multistatic radar, microwave tomography, 3D imaging, synthetic aperture radar

## Abstract

The advent of multichannel ground-penetrating radar systems capable of acquiring multiview, multistatic, and multifrequency data is offering new possibilities to improve subsurface imaging performance. However, this raises the need for reconstruction approaches capable of handling such sophisticated configurations and the resulting increase in the data volume. Therefore, the challenge lies in identifying proper measurement configurations that balance image quality with the complexity and duration of data acquisition. As a contribution to this topic, the present paper focuses on a measurement system working in reflection mode and composed of an array of antennas, consisting of a transmitting antenna and several receiving antennas, whose spatial offset is comparable to the probing wavelength. Therefore, for each position of the transmitting antenna, a single-view/multistatic configuration is considered. The imaging task is solved by adopting a linear microwave tomographic approach, which provides a qualitative reconstruction of the investigated scenario. In particular, a 3D inverse scattering problem is tackled for an isotropic, homogeneous, lossless, and non-magnetic medium under the Born approximation, considering both single- and multi-frequency data. A preliminary analysis, referring to a 3D free-space reference scenario, is performed in terms of the spectral content of the scattering operator and the system’s point spread function. Finally, an experimental validation under laboratory conditions is presented in order to verify the expected imaging capability of the inversion approach.

## 1. Introduction

Ground-Penetrating Radar (GPR) is a non-invasive sensing technology widely used in various fields, including environmental monitoring and civil engineering [1,2,3,4,5], archaeological investigations [6,7,8,9], agriculture [10,11], landmine detection [12,13], and planetary exploration [14,15,16]. Common GPR systems exploit a pair of antennas, one for transmission and one for reception, to send electromagnetic pulses into the subsurface and collect the echoes reflected by buried objects along a measurement line. However, in recent years, growing attention has been devoted to the development of multichannel systems [17,18,19,20,21,22,23,24]. In fact, due to their capability of exploiting multiple antennas for simultaneous transmission and reception, multichannel GPR systems allow for gathering an increased amount of data, with a positive impact on imaging results in terms of spatial resolution, robustness to signal interference, clutter suppression, target discrimination, and an extended detection range [25]. The effective exploitation of these new generation GPRs requires criteria to properly design the measurement configuration, i.e., to select a non- or minimally redundant number and position of the transmitting and receiving antennas and develop advanced signal processing strategies capable of fully exploiting the angular diversity offered by these multistatic and multiview setups [26].

Several measurement configurations and data processing strategies have been investigated in the literature [25,26]. Theoretically, GPR imaging involves the solution of a non-linear and ill-posed electromagnetic inverse scattering problem [26,27,28]. However, to make the imaging problem feasible in real conditions while still retaining relevant physical insights, a linearization of the inverse scattering problem is commonly performed by adopting a simplified model of the electromagnetic scattering phenomenon, for instance, that given by the Born approximation. Furthermore, a regularization technique is exploited to achieve a physically meaningful and stable solution [25,29]. It is worth pointing out that, due to model error introduced by the approximation, the linearization of the inverse problem allows the attainment of a qualitative image of the investigated scenario, in terms of location and shape of the buried targets. Although a quantitative estimate of the electromagnetic parameters of the targets is not possible, formulating the imaging as a linear inverse scattering problem under the Born approximation provides sufficient information in many GPR surveys [25,30]. Furthermore, it allows for a theoretical assessment of the imaging performance. In particular, the regularized point spread function (PSF) and spectral content (SC) are widely assessed theoretical figures of merit to predict reconstruction capabilities for a given measurement configuration (e.g., see [25,31]). Understanding how measurement configuration affects imaging performance is a crucial issue, as it enables the prediction of reconstruction quality for a given setup and, at the same time, provides guidelines for choosing the most appropriate configuration based on the electromagnetic characteristics of the investigated scenario [25,31].

The paper contributes to this topic and presents an analysis focused on SC and PSF for a bistatic measurement configuration operating in reflection mode. The imaging is formulated for a 3D scenario by using a linearized Microwave Tomographic (MWT) approach based on the Truncated Singular Value Decomposition (TSVD). This approach has been proposed and experimentally validated against monostatic GPR data in [32].

It is timely to note that previous studies in this field have mainly examined 2D scalar problems, providing valuable insights into the roles that illumination and frequency diversity play in imaging performance [31,33,34,35]. Extensions to 3D imaging have been considered in [36] and more recently in [23,37]. The aforementioned works have outlined the inherent filtering due to the regularization necessary to counteract the ill-posedness of the inverse scattering problem, which impairs the possibility of fully retrieving the target’s geometry [31,34,37]. In detail, for a reflection configuration typical of GPR applications, a low-pass filtering effect along the measurement direction and a band-pass filtering along the depth are observed. This means that only the upper and lower boundaries of a buried anomaly are typically estimated in tomographic images [31,34,37]. Furthermore, although the upper interface is accurately localized, the lower (deeper) boundary of the target is generally defocused and delocalized due to the fact that the propagation of the probing wave within the object is assumed to have the same electromagnetic velocity as that in the background medium [31,34]. Moreover, previous investigations have shown that a multimonostatic, multi-frequency configuration offers a good trade-off between image quality and measurement simplicity [31]. In contrast, multiview, multistatic, and multi-frequency acquisitions can further mitigate image degradation caused by suboptimal antenna spacing. In such cases, reducing the number of antennas can strike a trade-off between improving reconstruction quality and limiting system complexity [23].

This paper addresses the 3D reconstruction problem by considering a reflection measurement configuration composed of one transmitting (Tx) and four receiving (Rx) antennas, whose inter-element spacing is comparable to or greater than the probing wavelength. The system enables the acquisition of multibistatic (when a single receiving antenna is considered) and multistatic data (when multiple receiving antennas are exploited) for each location of the TX antenna. In particular, the present work analyzes the impact of both the number and spatial arrangement of Rx antennas on imaging performance, considering both single- and multi-frequency data. The SC of the scattering operator and the PSF are examined to assess the achievable imaging performance in free space, which is the simplest hosting medium. Consequently, 3D imaging is achieved using the approach previously proposed in [32], as adapted to the reference scenarios considered herein. Finally, a validation of the expected imaging capabilities is performed by processing experimental data collected at the Georgia Institute of Technology, Atlanta, USA (see [24]).

The paper is organized as follows. Section 2 recalls the mathematical formulation of the inverse scattering problem. Section 3 deals with the assessment of reconstruction capabilities. Section 4 presents the reconstruction results from the experimental dataset. Concluding remarks are summarized in Section 5.

## 2. GPR Imaging Approach

**Figure 1:**
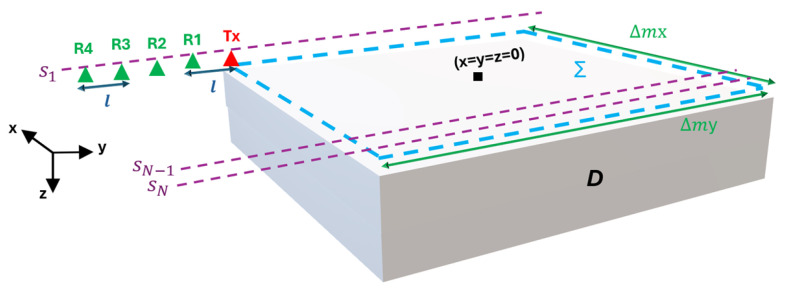
Reference scenario.

The geometry of the 3D imaging problem at hand is illustrated in Figure 1. The investigation domain D is probed by means of a system that exploits one Tx antenna and one or more (up to four) Rx antennas. As shown in Figure 1, the Rx antennas are positioned at progressively increasing offsets from the Tx one along the *y*-axis, and the offset of the *n*-th receiver is equal to nl, n=1,…,4, where *l* is the inter-element spacing. All antennas are located in the plane z=0 and are jointly moved at *N* discrete positions with a uniform spatial step $d_{m}$, along the *x* and *y* axes. The initial and final position of the TX antenna in the plane z=0 define the scanning planar surface Σ having size $\Delta_{mx}\times \Delta_{my}$, where $\Delta_{mx}=\Delta_{my}=\left(N-1\right)d_{m}$. Three measurement configurations are considered:

$$\left\{\begin{array}{cc} TR_{1}: & 1\;\mathrm{Tx},\;1\;\mathrm{Rx}\;\mathrm{with}\;\mathrm{offset}\;l\;\\ TR_{4}: & 1\;\mathrm{Tx},\;1\;\mathrm{Rx}\;\mathrm{with}\;\mathrm{offset}\;4l\;\\ TR_{1234}: & 1\;\mathrm{Tx},\;4\;\mathrm{Rx}\;\mathrm{with}\;\mathrm{offset}\mathrm{nl},n=1,\ldots ,4. \end{array}\right.$$

$TR_{1}$ and $TR_{4}$ are multibistatic configurations with the receiving antenna placed at different offsets from the Tx one. Moreover, $TR_{1234}$ is representative of a novel GPR configuration where, for each location of the TX antenna, the scattered field is collected by four receiving antennas ($R_{1}$, $R_{2}$, $R_{3}$, $R_{4}$) simultaneously.

As in [32], the imaging task is formulated as an inverse scattering problem in an isotropic, lossless, homogeneous, and non-magnetic medium ($\mu_{0}=4\pi \times 10^{-7}$ H/m). Furthermore, the Tx antenna is modeled as a *y*-directed Hertzian dipole, and the measured (scalar) quantity is assumed to be the *y*-component of the scattered field. The presence of targets in D is modeled by the unknown contrast function [38]:

(1) $$\chi \left(r\right)=\frac{\varepsilon \left(r\right)-\varepsilon_{b}}{\varepsilon_{b}}$$

where $\varepsilon_{b}$ is the background permittivity. Herein, we assume $\varepsilon_{b}=\varepsilon_{0}=8.85\times 10^{-12}$ F/m. Note that free space is taken as a preliminary hosting medium, properly representing an isotropic, homogeneous, lossless, and non-magnetic material. The targets are described by the permittivity function ε(r), where $r=x\hat{x}+y\hat{y}+z\hat{z}$ denotes a generic point in D.

According to the Born approximation, the relationship between the data, i.e., the *y*-component of the scattered electric field $E_{s}$ as measured at the point $r_{rx}=x_{rx}\hat{x}+y_{rx}\hat{y}$, and the unknown contrast χ, at the angular frequency ω, is defined as:

(2) $$E_{s}\left(r_{rx},r_{tx},\omega \right)=k^{2}\int_{D}\hat{y}\cdot \underline{\underline{G}}\left(r_{rx},r,\omega \right)\cdot {\underline{E}}_{\mathrm{inc}}\left(r,r_{tx},\omega \right)\;\chi \left(r\right)\;dr$$

where $k=\omega \sqrt{\mu_{0}\varepsilon_{0}}$ is the wavenumber in the background medium, $\underline{\underline{G}}$ denotes the dyadic Green’s function, and

(3) $${\underline{E}}_{\mathrm{inc}}\left(r,r_{tx},\omega \right)=-j\omega \mu_{0}I_{\Delta }\;\underline{\underline{G}}\left(r,r_{tx},\omega \right)\cdot \hat{y}$$

represents the incident electric field in D when the Tx antenna is located at $r_{tx}=x_{tx}\hat{x}+y_{tx}\hat{y}$, and $I_{\Delta }$ is the dipole current moment.

The dyadic Green’s function $\underline{\underline{G}}\left(r_{t},r,\omega \right)$ is expressed in the far-field region as [38]:

(4) $$\underline{\underline{G}}\left(r,r_{tx},\omega \right)=\frac{e^{-jkR_{tx}}}{4\pi R_{tx}}\left[\begin{array}{ccc} 1-\frac{{\left(x-x_{tx}\right)}^{2}}{R_{tx}^{2}} & -\frac{\left(x-x_{tx}\right)\left(y-y_{tx}\right)}{R_{tx}^{2}} & -\frac{(x-x_{tx})z}{R_{tx}^{2}}\\ -\frac{\left(x-x_{tx}\right)\left(y-y_{tx}\right)}{R_{tx}^{2}} & 1-\frac{{\left(y-y_{tx}\right)}^{2}}{R_{tx}^{2}} & -\frac{(y-y_{tx})z}{R_{tx}^{2}}\\ -\frac{(x-x_{tx})z}{R_{tx}^{2}} & -\frac{(y-y_{tx})z}{R_{tx}^{2}} & 1-\frac{z^{2}}{R_{tx}^{2}} \end{array}\right]$$

where $R_{tx}=\left|r-r_{tx}\right|=\sqrt{{\left(x-x_{tx}\right)}^{2}+{\left(y-y_{tx}\right)}^{2}+z^{2}}$ is the distance between r and $r_{tx}$. Note that, due to reciprocity, the Green’s function $\underline{\underline{G}}\left(r_{rx},r,\omega \right)$ appearing in (2) is defined by (4) with the only difference being the substitution of $r_{tx}$ with $r_{rx}$.

According to (2), the relationship between the scattered field data collected by the set of active receivers and the unknown contrast function χ is rewritten in a compact way as:

(5) $$E_{s}=A\chi$$

where $A:L^{2}\left(D\right)\to L^{2}\left(\Omega \right)$ is an integral operator mapping the unknown space of the contrast functions in the scattered field data space, $L^{2}$ being the space of square-integrable functions.

Since the operator A is compact, the inverse scattering problem defined in (5) is ill-posed [29]. Consequently, a regularization technique is required to achieve a stable and meaningful reconstruction. In this work, the Truncated Singular Value Decomposition (TSVD) [29] inversion scheme is exploited to achieve the regularized reconstruction as

(6) $${\bar{\chi }}_{R}\left(r\right)=\sum_{n=0}^{N_{\delta }}\frac{\langle E_{s},u_{n}\rangle }{\sigma_{n}}v_{n}\left(r\right).$$

In (6), $u_{n}$ and $v_{n}$ are the left and right singular functions that span the data and unknown spaces, respectively; ${\left\{\sigma_{n}\right\}}_{n=0}^{\infty }$ are the singular values of A ordered in decreasing magnitude, and $N_{\delta }$ is the truncation index, which is the regularization parameter to be fixed in order to achieve a strong compromise between accuracy and stability of the solution [27].

It is worth remarking that the electromagnetic scattering model error introduced by the Born approximation prevents any quantitative reconstruction. Specifically, the dielectric contrast given by (6) provides a qualitative estimate giving information about the target location and rough information about its size and shape. For this reason, the amplitude of the retrieved dielectric contrast, as normalized to its maximum value, is considered and referred to as the microwave tomographic (MWT) reconstruction of the scenario under test.

## 3. Imaging Performance Analysis

This section investigates the impact of the measurement configurations presented in Section 2 on the achievable imaging capabilities. This analysis is performed by resorting to two key figures of merit, namely the regularized PSF and SC [25].

The regularized PSF is the reconstruction of a point-like target, here achieved via TSVD. The examination of the PSF allows for estimating how the spatial resolution limits vary depending on the target position in the investigation domain D. The TSVD-based PSF is defined as follows:

(7) $$PSF\left(r\right)=\sum_{n=1}^{N_{\delta }}v_{n}^{*}\left(r^{\prime }\right)v_{n}\left(r\right),$$

where $r^{\prime }$ indicates the position of the point target and ^*^ denotes the complex conjugate operation.

The SC provides information about the spatial filtering performed by the scattering operator, allowing the “visualization” of the global harmonic content retrievable from the collected data. The SC is evaluated by considering all possible positions of a point target within the investigation domain *D* (see [39] for a detailed explanation). In particular, under the TSVD inversion strategy, SC is defined as the sum of the square moduli of the spectra of the singular functions $v_{n}$ for the chosen regularization parameter $N_{\delta }$ [25,31,35,36]:

(8) $$SC\left(k_{x},k_{y},k_{z}\right)=\sum_{n=1}^{N_{\delta }}{\left|{\hat{v}}_{n}\left(k_{x},k_{y},k_{z}\right)\right|}^{2}$$

In the previous equation, ${\hat{v}}_{n}\left(k_{x},k_{y},k_{x}\right)$ denotes the 3D Fourier Transform of $v_{n}\left(x,y,z\right)$, and $k_{x}$, $k_{y}$, $k_{z}$ are the spectral variables corresponding to *x*, *y*, and *z*, respectively.

The PSF and SC analysis allows for a comprehensive understanding of the imaging performance from a local and global perspective, respectively. On the other hand, the analysis is strictly related to the considered investigated scenario and measurement configuration because the scattering operator, and thus the PSF and SC, depend on them.

In the following, a numerical analysis is performed to compare SC and PSF for the three measurement configurations introduced in Section 2, considering single- and multi-frequency data. The comparison between the first two multibistatic setups ($TR_{1}$ and $TR_{4}$) provides insights into the effect of the transmitter–receiver offset on the imaging capabilities. In addition, the third setup based on four receivers ($TR_{1234}$) allows for assessing the impact of using more Rx antennas.

The numerical analysis is carried out over the frequency band B=[2200,5200] MHz, discretized with a step of 300 MHz, which is considered for the multi-frequency case. For the single-frequency scenario, the central frequency $f_{c}=3700$ MHz of the band is used, corresponding to a wavelength of $\lambda_{c}=0.08\;m$. The investigation domain is defined as $D=\left[-0.3\;,\;0.3\right]\times \left[-0.3\;,\;0.3\right]\times \left[0.3\;,\;0.62\right]\;m^{3}=\left[-3.7\lambda_{c}\;,\;3.7\lambda_{c}\right]\times \left[-3.7\lambda_{c}\;,\;3.7\lambda_{c}\right]\times \left[3.7\lambda_{c}\;,\;7.7\lambda_{c}\right]$, and discretized into cubic voxels with a side length of 1.25 cm (i.e., $\lambda_{c}/6.5$). The Tx scanning surface is expressed as $\Sigma =\left[-0.3\;,\;0.3\right]\times \left[-0.3\;,\;0.3\right]\;m^{2}=\left[-3.7\lambda_{c}\;,\;3.7\lambda_{c}\right]\times \left[-3.7\lambda_{c}\;,\;3.7\lambda_{c}\right]$, and it is sampled uniformly with a step size of $d_{m}=0.02\;m=\frac{\lambda_{c}}{4}$ along both the x- and y-directions. The inter-element spacing between adjacent antennas is $l=0.12\;m=1.5\;\lambda_{c}$. Since this spacing is larger than $\lambda_{c}$, the distance between the Tx and Rx antennas is not negligible and must be explicitly taken into account in the inversion process.

**Figure 2:**
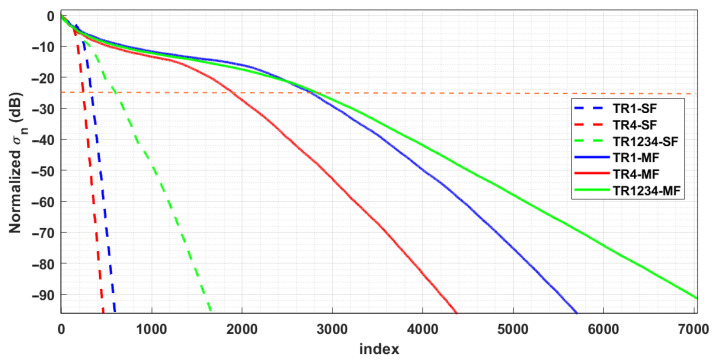
Normalized singular values (dB) for the considered measurement configurations. The dashed curves correspond to the single-frequency case, while the solid curves are relevant to the multi-frequency case.

Figure 2 illustrates the behavior of the normalized singular values of the operator A for all six measurement configurations. The graph reveals that the number of singular values changes significantly if single- or multi-frequency data are considered for a specific truncation level. As expected, for a fixed configuration, the number of significant singular values is notably larger in the multi-frequency case due to the increased information content of the data (e.g., see [25,31,36]) resulting from the exploitation of the working frequency band.

On the other hand, it is interesting to observe that, in both single- and multi-frequency cases, the multibistatic configuration $TR_{1}$ setup retains more significant singular values compared to the configuration $TR_{4}$.

The analysis of SC and PSF, defined by Equations (8) and (7), respectively, is performed here by setting the TSVD threshold to discard singular values 25 dB lower than the maximum one (horizontal line in Figure 2).

**Figure 3:**
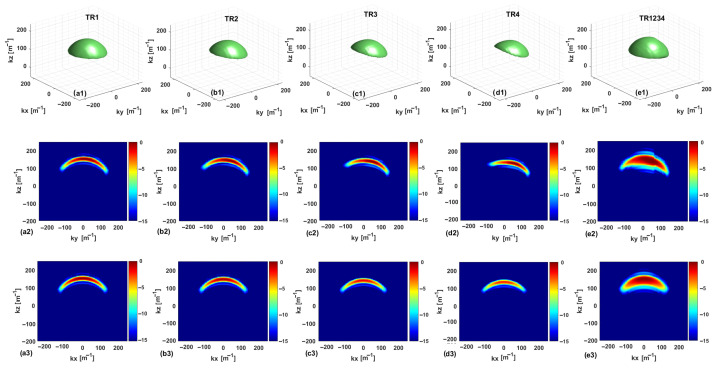
Normalized spectral content (dB) in the single-frequency case. The first row (**a1**–**e1**) is an isosurface representation with a threshold of −7 dB. The second and third rows (**a2**–**e2**,**a3**–**e3**) display the cuts in the planes $k_{x}=0$ and $k_{y}=0$, respectively.

**Figure 4:**
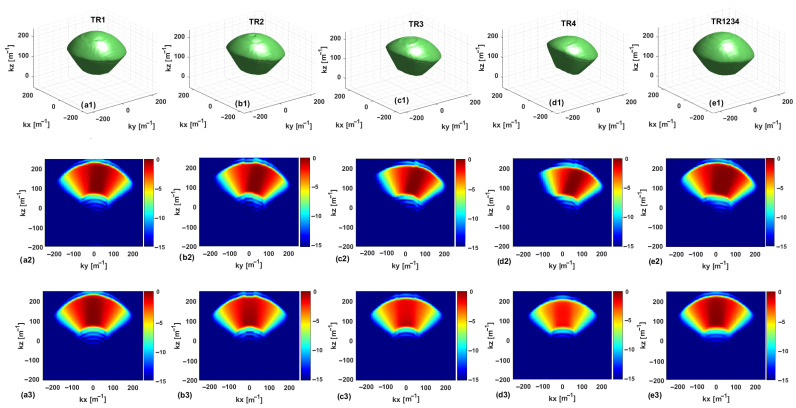
Normalized spectral content (dB) in the multi-frequency case. The first row (**a1**–**e1**) is an isosurface representation with a threshold of −7 dB. The second and third rows (**a2**–**e2**,**a3**–**e3**) display the cuts in the planes $k_{x}=0$ and $k_{y}=0$, respectively.

The SC plots are shown in Figure 3 and Figure 4 for the single- and multi-frequency cases, respectively. To improve clarity and understanding, the multibistatic configurations $TR_{2}$ and $TR_{3}$, corresponding to offset values equal to 2l and 3l, respectively, are considered in addition to the above-mentioned configurations $TR_{1}$, $TR_{4}$, and $TR_{1234}$. For a consistent comparison, each graph is normalized to its respective maximum. In each figure, the first row presents isosurface plots of the SC when the visualization threshold is set equal to −7 dB. The second and third rows show the SC cuts in the planes $k_{x}=0$ and $k_{y}=0$, respectively. Furthermore, the first, second, third, fourth, and fifth columns correspond to $TR_{1}$, $TR_{2}$, $TR_{3}$, $TR_{4}$, and $TR_{1234}$, respectively.

By analyzing the SC for the single-frequency case, the cut at $k_{x}=0$ shows an increased asymmetry when passing from $TR_{1}$ to $TR_{4}$. Note that the asymmetry along $k_{y}$, already present for $TR_{1}$, is more and more evident for the cases $TR_{2}$, $TR_{3}$, and $TR_{4}$. This asymmetry is due to the asymmetric arrangement along *y* of the Rx measurement points concerning the $T_{x}$ antenna (all the RX antennas are positioned on the same side relative to the TX antenna). The inter-element spacing increases by turning from $TR_{1}$ to $TR_{4}$, and for $TR_{4}$, it is larger than the semi-extent of the investigation domain along the *y*-axis. In conclusion, a significant asymmetry of the Rx locations is observed, since at the extremes of the measurement domain, Rx antennas occupy the locations $-\Delta_{my}/2-nl$ and $\Delta_{my}/2-nl$ for n=1,2,3,4. This is reflected in an asymmetry of the support of the SC domain along $k_{y}$. In both multibistatic cases, the SC has very tight support along $k_{z}$, which entails that the expected resolution limits along the depth are very limited. By looking at the SC for the two multibistatic cases, the support of SC is smaller for the configuration $TR_{4}$, which is also indicative of the fact that the number of singular values retained in the TSVD expansion is smaller compared to the case of the $TR_{1}$ configuration.

The situation is different for the multistatic case $TR_{1234}$, where the SC can be approximately assumed to be the superposition of the ones corresponding to the four multibistatic cases. In the multistatic case, we still have an asymmetry along the $k_{y}$ axis and a thicker support of the SC along $k_{z}$, which corresponds to an improved resolution along the *z*-axis. Along the $k_{x}$ axis, a spectral content similar to that of a multimonostatic configuration is achieved for the three configurations. This is justified by the fact that all the antennas are at the same coordinate along the *x*-axis. It is worth noting that, for the $TR_{4}$ configuration, SC does not attain its maximum over the cut at $k_{y}=0$ due to the asymmetry along the $k_{y}$ axis.

**Figure 5:**
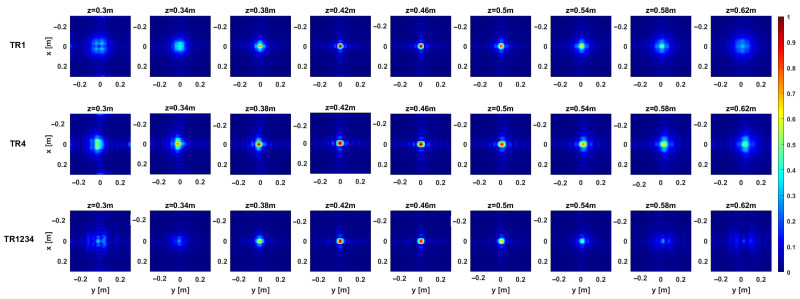
Depth slices of the normalized PSF amplitude for a point-like target located at (0,0,0.46) m for the considered configurations in the single-frequency case.

**Figure 6:**
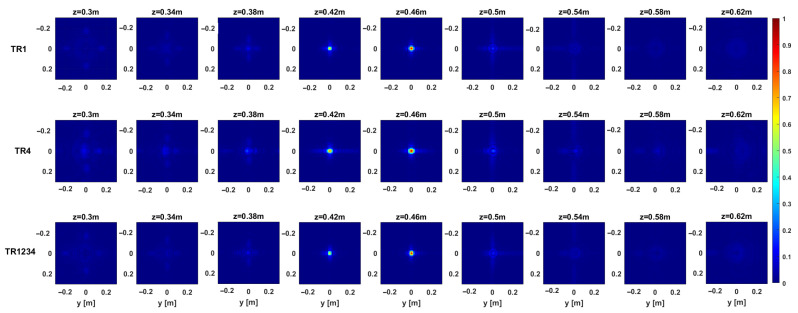
Depth slices of the normalized PSF amplitude for a point-like target located at (0,0,0.46) m in the multi-frequency case.

**Figure 7:**
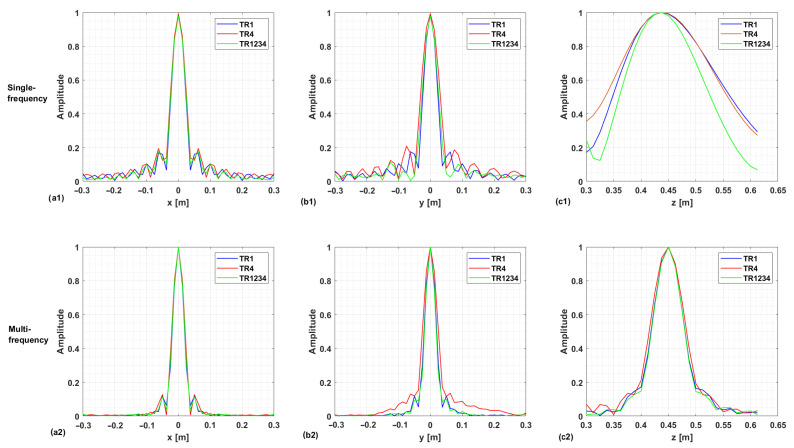
Comparison of the normalized PSF cuts corresponding to a point-like target located at (0,0,0.46) m. Panels (**a**–**c**) show the PSF cuts along the *x*, *y*, and *z*-axes, respectively. The first row corresponds to the single-frequency case, while the second row pertains to the multi-frequency case.

The above considerations are confirmed by the regularized PSF referring to a point target located at the center of the investigation domain at (0 , 0, 0.46)m. The PSF amplitude, as normalized to its maximum value, is illustrated in Figure 5 and Figure 6 for each configuration in the single-frequency and multi-frequency cases, respectively. Moreover, Figure 7 compares the normalized PSF cuts along the *x*, *y*, and *z* directions for the single-frequency and multi-frequency cases. For the single-frequency case, by examining Figure 5 and the upper row of Figure 7, we can observe that the regularized PSF is quite similar along *x*- and *y*-axes for the three considered configurations $TR_{1}$, $TR_{4}$, and $TR_{1234}$. The situation is different for the behavior of the PSF along the *z*-axis, where we observe an improved resolution in the multistatic case. Table 1 summarizes the resolution values along the *x*, *y*, and *z* directions computed as the full-width at half maximum (FWHM) of the PSF main lobe. For the single-frequency case, the table highlights the improved resolution limits along the *z*-axis for the multistatic case. Conversely, the difference among the three configurations outlined above in the single-frequency case almost disappears in the multi-frequency case.

As a result, if adding more receivers is expected to enhance the imaging performance in the single-frequency case, the use of more than one receiver does not affect resolution capabilities when multi-frequency data are collected. In other words, the information provided by multistatic diversity is not completely independent from the multi-frequency one, as formerly observed in relation to the investigation of pillars [39]. For the case at hand, the above statement is confirmed by the fact that the singular value curves, the spectral contents, and the regularized PSFs for the cases $TR_{1}$ and $TR_{1234}$ are very similar. This entails that the receivers 2, 3, and 4 do not add a significant amount of independent information in the multi-frequency case. It is worth noting that the resolution limits along the *z*-axis are dictated by the working frequency band, and a substantial improvement of the spatial resolution along the *z*-axis is observed, thus allowing the target localization.

## 4. Experimental Results

This section presents experimental imaging results obtained for different types of targets. The scattered field data have been obtained from laboratory tests carried out in past years at the Georgia Institute of Technology, Atlanta, USA, and made available to the scientific community [24]. The data were collected by means of a vector network analyzer in the frequency range [600, 8600] MHz with a step of 20 MHz (401 frequency samples), using two Tx antennas ($T_{x1}$ and $T_{x2}$) and four Rx antennas. The spacing of the *n*-th Rx antenna from the Tx one is nl with n=1,…,4 and $l=0.12\;m=1.5\;\lambda_{c}$. All antennas are deployed at the same height, aligned along the *y*-axis in the *x*-*y* plane, and moved by means of a 3D positioner with a step of $0.48\;m=6\;\lambda_{c}$ in the *x* and *y*. Additional details about the measurement instrumentation and experimental conditions can be found in [24].

**Figure 8:**
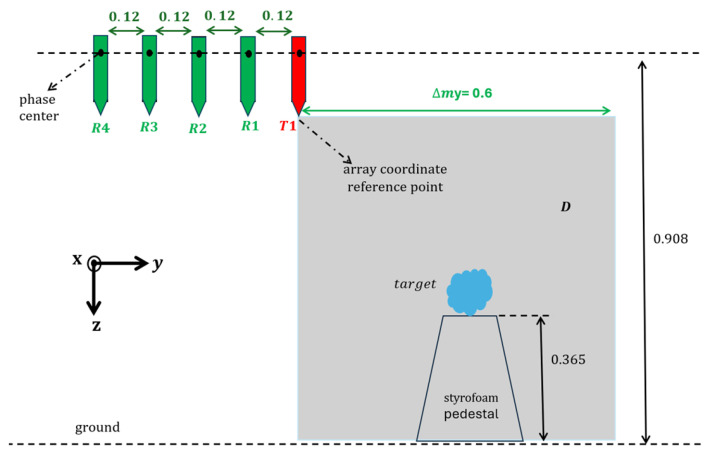
View of the experimental setup in the *y*-*z* plane. The distances are expressed in meters.

In this work, we process experimental data collected by the transmitter $T_{x1}$ for targets placed above the ground (i.e., in air). The numerical parameters D,Σ,l, and $d_{m}$ are the ones introduced in Section 3. The antennas are positioned $0.54\;m=6.8\;\lambda_{c}$ above a styrofoam pedestal (along the *z*-axis), where the targets are placed, as illustrated in Figure 8. This figure provides a 2D view of the experimental setup in the *y*-*z* plane.

**Figure 9:**
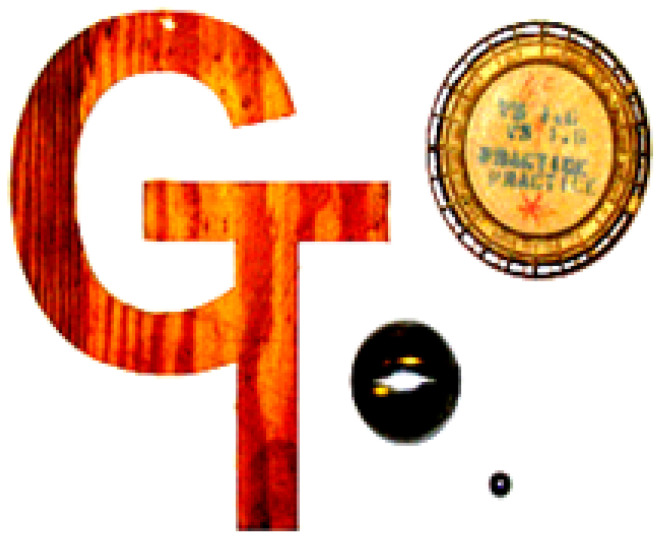
Picture of the considered targets [24].

As shown in Figure 9, four different targets are considered:

- Target 1: Metallic sphere having a diameter of 2 cm;
- Target 2: GT-shaped plywood object with a thickness of 1.8 cm;
- Target 3: Metallic sphere having a diameter of 11 cm;
- Target 4: VS-1.6 anti-tank mine replica, with a diameter of 22 cm and a height of 9.2 cm.

A single dataset was collected for each target, which, individually, was placed on the styrofoam pedestal before starting the data acquisition. The styrofoam was 0.365 m high and centered relative to the measurement surface Σ.

### 4.1. Data Processing

The frequency domain raw data account for direct antenna coupling, the target response, and clutter from the surrounding environment, such as reflections from the ground. These data were originally calibrated by the authors in [24] to compensate for propagation delays (zero-time setting) and attenuation in cables, and eliminate the direct coupling between the Tx and Rx antennas.

**Figure 10:**
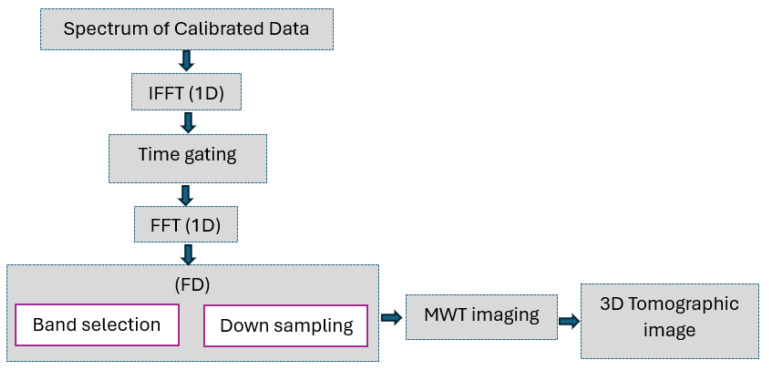
GPR data processing flowchart.

The adopted data processing pipeline is summarized by the flowchart reported in Figure 10. First, a 1D Inverse Fast Fourier Transform (IFFT) is applied to convert the calibrated frequency data collected by each receiver into the time domain. Following this, a time gating is performed to suppress dominant signal reflections from the ground. The gating time $t_{g}$ needs to be lower than the arrival time related to the ground reflection at the closest receiver $\left(R_{x1}\right)$, which is expressed as:

(9) $$t_{g}^{*}=2\frac{\sqrt{\Delta z^{2}+{\left(r_{1}/2\right)}^{2}}}{c}$$

where Δz represents the distance between Σ and the ground (i.e., 0.908 m) and $r_{1}=0.12$ m is the distance between $T_{x1}$ and the first receiving antenna. Based on Equation (9), a value $t_{g}^{*}=6.07$ ns is obtained, and a more conservative choice is made by setting the gating time as $t_{g}=4.87$ ns, i.e., by setting to zero all the time-domain data at a fast time larger than 4.87 ns. After time gating, the signal is transformed again into the frequency domain over the band B=[2200,5200] MHz, which is sampled with a step of 300 MHz before performing MWT imaging.

**Figure 11:**
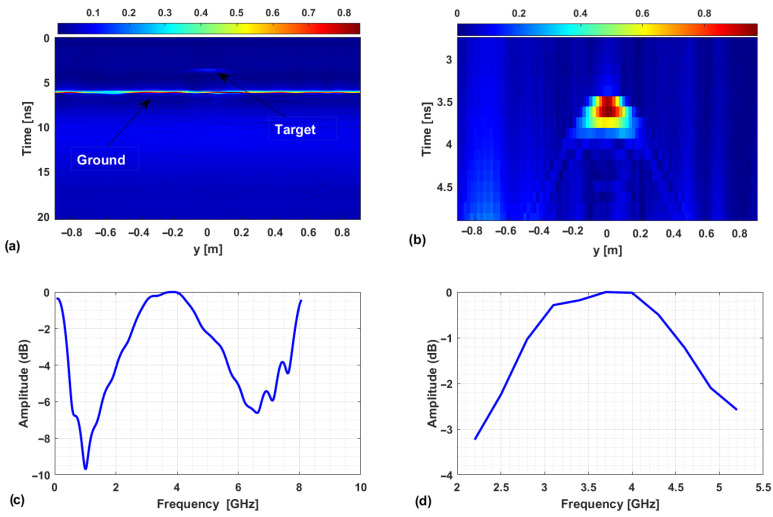
Experimental data for Target 1. (**a**) Calibrated data in the time domain. (**b**) Filtered data after time gating. (**c**) Average spectrum along the *y*-axis. (**d**) Average spectrum within the selected bandwidth used for imaging.

Figure 11 illustrates the effect of the processing steps before MWT imaging. The data shown are multibistatic/multi-frequency and obtained by considering the setup $T_{R1}$ and Target 1. The panel a) shows the amplitude of calibrated data in the time domain at x=0 m. As expected, they contain various signal components: a strong reflection from the ground surface and the signal due to the target. The image in panel b) shows the corresponding results after applying time gating. In this case, the target signal contribution becomes clearly detectable. Panel c) displays the amplitude of the data spectrum averaged over the measurement points along the *y*-axis. The curve reveals the overall spectral behavior of the signal, highlighting the most significant frequency components. The graph in panel d) illustrates the amplitude of the final spectrum following bandwidth selection and frequency downsampling. Specifically, the number of frequency samples has been reduced from 401 (panel c) to 11 (panel d), while retaining the essential spectral components. This reduced dataset significantly lowers the computational burden of the imaging algorithm in the multi-frequency case, without compromising the reconstruction accuracy.

### 4.2. Reconstruction Results

The MWT approach described in Section 2 is applied to reconstruct the geometrical features of the targets. Following the approach in Section 3, we evaluate the imaging performance of the considered measurement configurations using real data. The comparison is carried out in the case of Target 1, considering both single- and multi-frequency data. Owing to its small size, Target 1 can be reasonably approximated as a point-like target, thus enabling a meaningful experimental assessment of the resolution limits.

**Figure 12:**
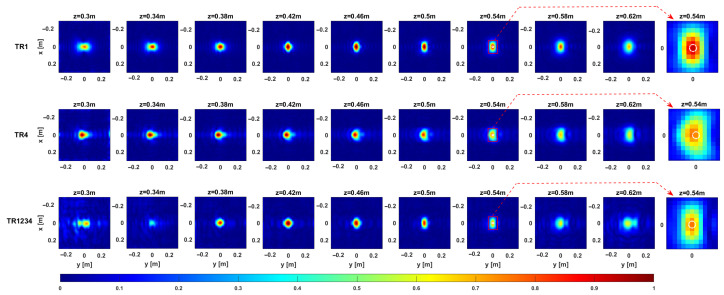
Target 1. Depth slices of the 3D MWT reconstruction. The first, second, and third rows correspond to configurations $TR_{1}$, $TR_{4}$, and $TR_{1234}$, respectively, for the single-frequency case. The amplitudes are normalized with respect to their maximum value in the volume. The white dashed line shows the right location of the target. The right side panel represents the zoom around the target of the cut at z=0.54 m.

**Figure 13:**
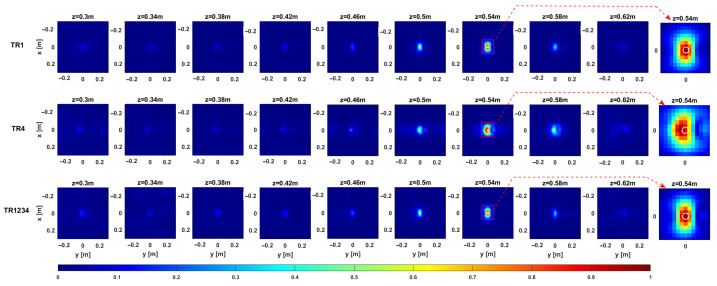
Target 1. Depth slices of the 3D MWT reconstruction. The first, second, and third rows correspond to configurations $TR_{1}$, $TR_{4}$, and $TR_{1234}$, respectively, for the multi-frequency case. The amplitudes are normalized with respect to their maximum value in the volume. The dashed white line shows the right location of the target. The right side panel represents the zoom around the target of the cut at z=0.54 m.

Figure 12 and Figure 13 provide representative depth slices of the tomographic reconstructions for single-frequency and multi-frequency configurations, respectively. As expected, the availability of multi-frequency data allows for significantly better resolution along the depth (range) for each configuration. Moreover, if the $TR_{1234}$ setup performs slightly better than $TR_{1}$ and $TR_{4}$ along depth for the single-frequency case, all setups yield nearly identical results in the multi-frequency case, with a resolution limit of approximately 5 cm. Furthermore, increasing the Tx-Rx offset yields slightly worse resolution along the *y*-axis, as previously observed in Section 3.

In order to perform a quantitative comparison among the MWT reconstructions, the Root Mean Square (RMS) contrast [40] metric is employed:

(10) $$C_{rms}=\sqrt{\frac{1}{Q}\sum_{q=1}^{Q}{\left({\bar{\chi }}_{q}-{\bar{\chi }}_{avg}\right)}^{2}}$$

where *Q* denotes the total number of pixels in the investigation domain D, and ${\bar{\chi }}_{q}$ and ${\bar{\chi }}_{avg}$ represent the amplitude of the *q*-th pixel and the mean amplitude of the reconstructed contrast function, respectively. The $C_{rms}$ metric decreases as the tomographic image exhibits improved focusing quality. Therefore, lower $C_{rms}$ values indicate better reconstruction performance. Table 2 provides the $C_{rms}$ values for all the extended targets considered herein. The $C_{rms}$ values show a strong improvement in reconstruction, focusing on the multi-frequency case compared to the single-frequency one. Furthermore, in the multi-frequency case and for all the targets, the $C_{rms}$ values corresponding to $TR_{1}$ and $TR_{1234}$ are (sometimes) identical to each other and lower than those of $TR_{4}$. This outcome indicates that a larger inter-element spacing between the transmitting and receiving antennas leads to poorer reconstruction results. The findings confirm again that, although in the single-frequency scenario the use of multiple receivers can slightly improve the reconstruction quality, in the multi-frequency scenario this advantage is not observed when compared to the results of the $TR_{1}$ configuration.

The above results demonstrate that the $TR_{1}$ configuration provides the best trade-off between efficiency and resolution in the multi-frequency case, thus confirming the analysis of the imaging capabilities in the previous section. Accordingly, and for brevity, the configuration $TR_{1}$ is the only one considered from this point on.

**Figure 14:**
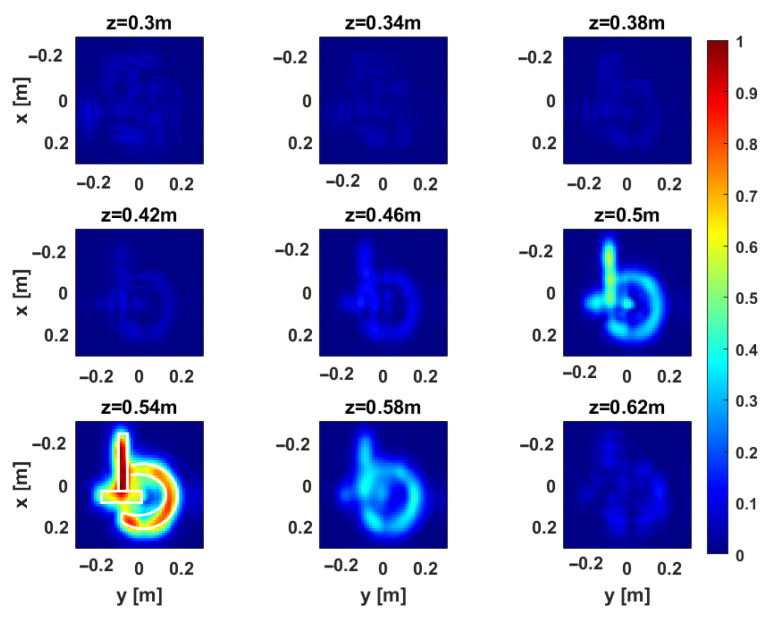
Target 2. Depth slices of the 3D MWT reconstruction obtained with $TR_{1}$ configuration for the multi-frequency case. The amplitudes are normalized with respect to their maximum value in the volume. The dashed white line shows the right location of the target.

**Figure 15:**
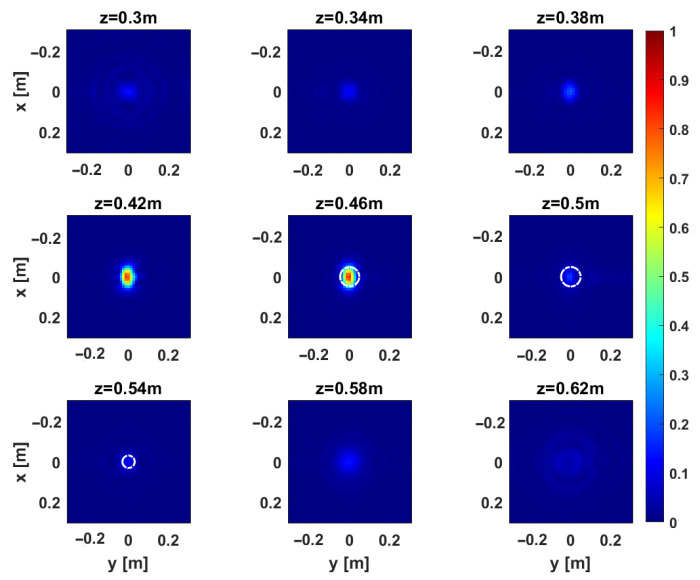
Target 3. Depth slices of the 3D MWT reconstruction obtained with $TR_{1}$ configuration for the multi-frequency case. The amplitudes are normalized with respect to their maximum value in the volume. The dashed white lines show the right location of the target.

Figure 14 and Figure 15 present the depth slices of the 3D MWT reconstructions for Target 2 (GT-shaped plywood) and Target 3 (0.11 m diameter metallic sphere), respectively. The GT-shaped target is more challenging due to its complex geometry, making it an effective benchmark for the reconstruction capabilities of the $TR_{1}$ configuration. As shown in Figure 14, the shape is accurately recovered, demonstrating the method’s effectiveness and reliability. As for the metallic sphere in Figure 15, the reconstruction reveals a pronounced peak corresponding to its top surface, confirming that the system has a strong resolving power along both depth and transverse directions.

**Figure 16:**
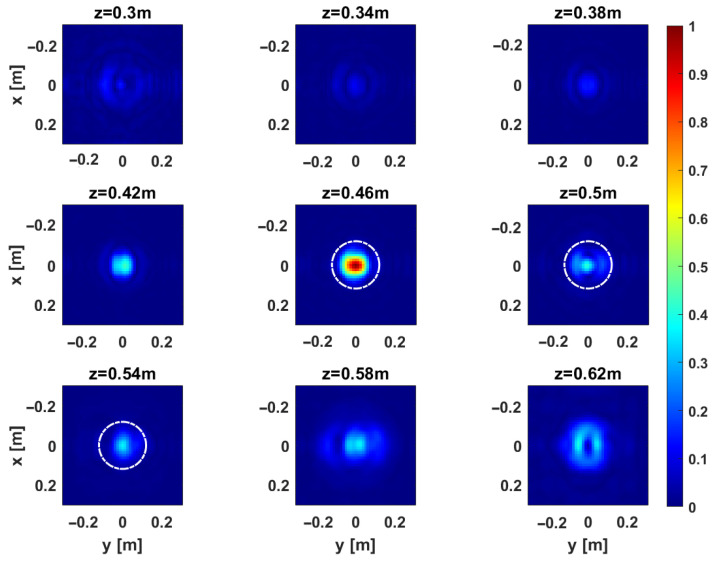
Target 4. Depth slices of the 3D MWT reconstruction obtained with $TR_{1}$ configuration for the multi-frequency case. The amplitudes are normalized with respect to their maximum value in the volume. The dashed white lines show the right location of the target.

The imaging results for Target 4 (VS-1.6 anti-tank mine) exhibit a more complex behavior, as shown in Figure 16. This complexity arises from the heterogeneous internal structure of the mine, which consists of materials with varying dielectric properties. Specifically, the outer surface is composed of a low-permittivity material (approximately $\varepsilon_{r}\approx 3$), while the explosive charge and certain internal components exhibit higher permittivity values (up to $\varepsilon_{r}\approx 5$). Consequently, the reconstructed image contains multiple spots; however, despite this, the transverse resolution remains satisfactory since the target is reliably identified along the *x* and *y* directions.

## 5. Conclusions

This paper investigated the effect of the measurement configuration on the spatial resolution achievable when employing a linear microwave tomographic approach. Two primary configurations were analyzed: bistatic and multistatic. In the bistatic setup, various transmitter–receiver offsets in the order of the probing wavelength were examined to evaluate their impact on image resolution, and the resulting performance was compared to that of the multistatic configuration.

The study considered both single- and multi-frequency data, and the reconstruction capabilities were assessed in terms of spectral content and the point spread function. Numerical outcomes, referred to a preliminary free-space scenario, showed that smaller transmitter–receiver offsets in bistatic configurations generally lead to an improved resolution. In the multistatic case, increasing the number of receiving antennas was found to partially compensate for limited frequency bandwidth, thereby enhancing image quality. On the other hand, in the multi-frequency case, the resolution enhancement provided by considering multiple receivers is almost negligible.

The theoretical findings were supported by experimental reconstructions of extended targets under laboratory conditions, which are of general interest for the case of a signal propagation occurring in an isotropic, homogeneous, and lossless medium under a reflection configuration. Future research activities will consider contact and contactless GPR scenarios and will address the effects of the radiation patterns of both transmitting and receiving antennas in the 3D case.

## Figures and Tables

**Table 1:** Resolution limits corresponding to the results in Figure 7 for the considered configurations in the single-frequency (SF) and multi-frequency cases (MF).

Resolution	${\mathrm{TR}}_{1}:\mathrm{SF}/\mathrm{MF}$	${\mathrm{TR}}_{4}:\mathrm{SF}/\mathrm{MF}$	${\mathrm{TR}}_{1234}:\mathrm{SF}/\mathrm{MF}$
Δx [m]	0.05/0.04	0.05/0.04	0.05/0.04
Δy [m]	0.05/0.04	0.06/0.05	0.05/0.04
Δz [m]	0.20/0.06	0.21/0.06	0.17/0.06

**Table 2:** Quantitative comparison of $C_{rms}$ values for different targets and configurations.

Targets	Target 1	Target 2	Target 3	Target 4
$TR_{1}$ (SF/MF)	0.08/0.03	0.20/0.10	0.08/0.03	0.12/0.06
$TR_{2}$ (SF/MF)	0.10/0.05	0.20/0.10	0.09/0.04	0.13/0.08
$TR_{1234}$ (SF/MF)	0.08/0.03	0.18/0.10	0.05/0.03	0.07/0.06

## Data Availability

The experimental data that support the findings of this study were collected at Georgia Institute of Technology, Atlanta, USA.
